# Risk of Epilepsy in Children Presenting to Emergency Departments with Their First Afebrile Seizure: A Retrospective Multicenter Study

**DOI:** 10.3390/children9111741

**Published:** 2022-11-12

**Authors:** Seungho Woo, Sangun Nah, Minsol Kim, Sangil Kim, Dongwook Lee, Jieun Moon, Sangsoo Han

**Affiliations:** 1 Department of Emergency Medicine, Soonchunhyang University Bucheon Hospital, Bucheon 14584, Korea; 2 Department of Pediatrics, Soonchunhyang University Bucheon Hospital, Bucheon 14584, Korea; 3 Department of Emergency Medicine, Soonchunhyang University Seoul Hospital, Seoul 04401, Korea; 4 Department of Emergency Medicine, Soonchunhyang University Cheonan Hospital, Cheonan 31151, Korea; 5 Department of Biostatistics, Clinical Trial Center, Soonchunhyang University Bucheon Hospital, Bucheon 14584, Korea

**Keywords:** seizure, epilepsy, age at onset, lactic acid, lethargy

## Abstract

Seizure is one of the most common neurologic disorders in pediatric emergency department visits. Early detection of epilepsy development in children with afebrile seizures is important. We identified predictors of epilepsy development in children with their first afebrile seizure. In this retrospective multicenter study, we enrolled pediatric patients aged 1 month to 18 years who presented with afebrile seizures at the emergency department from January 2017 to December 2020. Multivariable logistic regression analysis was performed to identify factors associated with epilepsy development. A total of 417 pediatric patients were enrolled, 161 (38.6%) of whom developed epilepsy. From the multivariable logistic regression analysis, older age at onset (2–5 years, odds ratio [OR] 2.611, *p* = 0.010; 11–15 years, OR 3.138, *p* = 0.003; 16–18 years, OR 4.292, *p* = 0.002), longer seizure duration of more than 10 min (OR 4.869, *p* = 0.006), two or more seizures (OR 2.378, *p* = 0.004), lethargy (OR 2.341, *p* = 0.021), and a lactate level > 2.27 mg/dL (OR 4.205, *p* < 0.001) were significant predictors for the development of epilepsy in children experiencing their first afebrile seizure.

## 1. Introduction

Epilepsy, which is a common, chronic, and serious brain neurological disorder, is characterized by persistent seizures [1,2]. Seizures account for about 1% of all emergency department (ED) visits and about 2% of all pediatric ED visits in the United States [3]. When a child presents to the ED with a seizure, it is first classified as febrile or afebrile based on whether the child experienced a fever within the last 24 h. This is important because the risk of seizure recurrence and epilepsy development is different among the two types of seizures [4]. Afebrile seizures are less common than febrile seizures but are much more likely to recur and progress to epilepsy [4,5,6].

Early detection of epilepsy development in children with afebrile seizures is important [1,7,8,9,10,11]. When evaluating a new-onset afebrile seizure in children, electroencephalography (EEG) and neuroimaging tests are recommended as part of the neuro-diagnostic evaluations [12,13]. However, it is difficult to perform routine EEG in the ED given the lack of equipment and staffing problems (EEG laboratories usually operate during the daytime) [14]. Emergency pediatric neuroimaging such as magnetic resonance imaging (MRI) is also difficult because most children require sedation and monitoring during examination [15].

Therefore, numerous studies have utilized factors that are readily available in EDs, such as seizure characteristics and laboratory results, to identify predictors of epilepsy development in children presenting with their first afebrile seizure. However, previous studies have presented a small sample size as a single study or only focused on seizure characteristics [16,17,18,19,20,21,22]. Our study was conducted as a multicenter study and considered various factors including symptoms, signs, and laboratory results of the patients. Moreover, we constructed a decision tree for physicians to predict the development of epilepsy among children with afebrile seizure earlier. Through this, physicians may provide early appropriate treatment to prevent or reduce adverse socio-environmental, psychological, and physical effects of epilepsy [7,8,9].

## 2. Materials and Methods

### 2.1. Study Design and Setting

In this retrospective multicenter study, we enrolled pediatric patients who presented with seizures at the ED from January 2017 to December 2020. Three EDs serve approximately 60,000, 50,000, and 40,000 patients annually, respectively, and are located in Gyeonggi, Chungcheong, and Seoul, Republic of Korea, respectively. The three EDs have shared one unified protocol for pediatric patients with afebrile seizures. According to the protocol, a fixed list of history taking (past history, seizure features, and symptoms), physical examinations (dehydrated tongue, neck stiffness, and Babinski sign), and blood tests (blood gas analysis, complete blood cell count [CBC], blood chemistry tests, C-reactive protein [CRP] levels) were conducted when a child first arrived. Then, most of the patients were hospitalized for further evaluation. Previously healthy children aged 1 month to 18 years, who had been afebrile for at least 24 h and presented with their first seizures, were enrolled. Patients with fever, previously diagnosed with epilepsy, with a recent trauma, or with incomplete medical records were excluded. The study was approved by our hospital Institutional Review Board (IRB file no. 2021-03-030, Approval date: 6 May 2021).

### 2.2. Patients and Data Collection

We extracted pediatric patients whose main symptom was seizure at the time of ED visit from electronic medical records. The medical records of 3074 patients, such as past history, seizure features, symptoms, physical examinations, and laboratory results, were reviewed by two experienced emergency physicians. The following data were extracted: age of first unprovoked seizure [16], sex, relevant medical history (birth details, neonatal intensive care unit admission, and family history), number of seizures within the 24 h after the first presentation [17], seizure duration [18], focal manifestations such as Todd’s paralysis, seizure type [19], postictal confusion, symptoms (headache, vomiting, diarrhea, poor oral intake [POI], and lethargy), physical examinations (dehydration, neck stiffness, and Babinski sign), and laboratory results (blood pH, bicarbonate, lactic acid [20], CBC, blood urea nitrogen, creatinine [21], glucose [22], albumin, aspartate aminotransferase [AST], alanine transaminase [ALT], sodium, chloride, total calcium, and CRP levels). All subjects were followed up for 1–5 years through a pediatric physician’s office or telephone interviews after the visit to the ED; development of epilepsy was determined by pediatric physician’s records or via telephone interviews.

### 2.3. Definitions of First Afebrile Seizure and Epilepsy

We defined a first afebrile seizure as a first-onset seizure without any acute provoking factors such as fever, in line with the definition of the International League against Epilepsy (ILAE) [23]. Epilepsy was defined as follows: at least two unprovoked seizures ≥ 24 h apart; one unprovoked seizure with a risk of further seizures similar to the general recurrence risk after two seizures (at least 60%) over the next 10 years; or diagnosis of an epilepsy syndrome using the ILAE definition [23].

### 2.4. Data Analysis

For all statistical analyses, SPSS (ver. 26,0; IBM Corp., Armonk, NY, USA) and R software (version 4.2.1; R Foundation for Statistical Computing, Vienna, Austria) were used. Categorical variables are expressed as absolute numbers (with percentages). Fisher’s exact test or the Pearson chi-squared test was used to compare categorical variables between two groups. The normality of the distribution of continuous variables was evaluated by the Shapiro-Wilk test. All continuous variables are expressed as medians with interquartile range, because all were not normally distributed. The Mann-Whitney U test was used to compare continuous variables between the two groups. Two-tailed *p*-values < 0.05 were considered statistically significant. Multivariable logistic regression analysis was performed on factors found to be statistically significant in univariable logistic regression analysis and variables reported to be significantly associated with epilepsy development in previous studies [6,16,17,18,19,20,21,22]. The Youden index was employed to determine the optimal cutoff values for continuous variables. Odds ratios (ORs) with 95% confidence intervals (CIs) were calculated via multivariable logistic regression analysis. The extent of multicollinearity was assessed by the variance inflation factor (VIF). The area under receiver operating characteristic curve (AUROC) was calculated when evaluating the results of multivariable logistic regression. Finally, a decision tree was constructed using independent prognostic factors identified by the multivariable logistic regression analysis results.

## 3. Results

**Figure 1:**
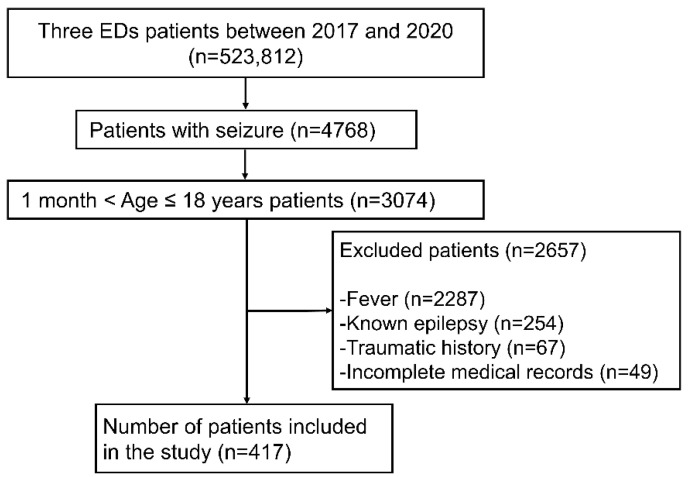
Flow chart of patient selection. ED, emergency department.

A total of 3074 pediatric seizure patients were enrolled during the study period, of whom 2657 were excluded because they had a fever (*n* = 2287), a history of seizure disorder (*n* = 254), trauma (*n* = 67), or incomplete medical records (*n* = 49). Ultimately, 417 pediatric patients with new-onset afebrile seizures were included in the study, of whom 161 developed epilepsy (Figure 1).

Table 1 summarizes the baseline characteristics of the non-epilepsy and epilepsy groups. There were no significant differences in sex, relevant medical history, postictal status, seizure type, symptoms (headache, vomiting, and POI), physical examinations, or various laboratory results (i.e., bicarbonate, CBC, bilirubin, AST, ALT, and chloride levels). The epilepsy patients were older than those without epilepsy (7 [3–13] vs. 6 [1.7–10] years, *p* = 0.015). In terms of seizure features, the epilepsy group experienced more seizures (1 [1,2] vs. 1 [1–1], *p* < 0.001), a higher rate of Todd’s paralysis (6.21% vs. 1.56%, *p* = 0.022), longer seizure duration (5 [2–10] vs. 3 [1–5] min, *p* < 0.001), a higher rate of lethargy (18.63% vs. 10.16%, *p* = 0.020), and a lower rate of diarrhea (3.73% vs. 10.16%, *p* = 0.027). The proportion of patients with dehydration was significantly higher in the non-epilepsy group (5.08% vs. 0.62%, *p* = 0.029). In terms of the laboratory results, the levels of lactate (2.7 [1.8–4.9] vs. 1.9 [1.4–2.6] mg/dL, *p* < 0.001), creatinine (0.5 [0.4–0.7] vs. 0.5 [0.4–0.6] mg/dL, *p* = 0.004), glucose (108 [97–127] vs. 100 [91–110] mg/dL, *p* < 0.001) and sodium (140 [138–141] vs. 139 [138–140] mmol/L, *p* = 0.005) were significantly higher in the epilepsy group, while the blood pH (7.36 [7.3–7.4] vs. 7.33 [7.2–7.4], *p* < 0.001) and BUN (11.6 [9.5–13.8] vs. 11 [9.0–13.2] mg/dL, *p* = 0.041), albumin (4.6 [4.4–4.8] vs. 4.5 [4.4–4.7] g/dL, *p* = 0.037), total calcium (9.6 [9.2–10] vs. 9.2 [8.9–9.6] mg/dL, *p* < 0.001), and CRP (1.6 [0.4–4.0] vs. 0.8 [0.3–3.8] mg/L, *p* = 0.033) levels were significantly higher in the non-epilepsy group.

**Figure 2:**
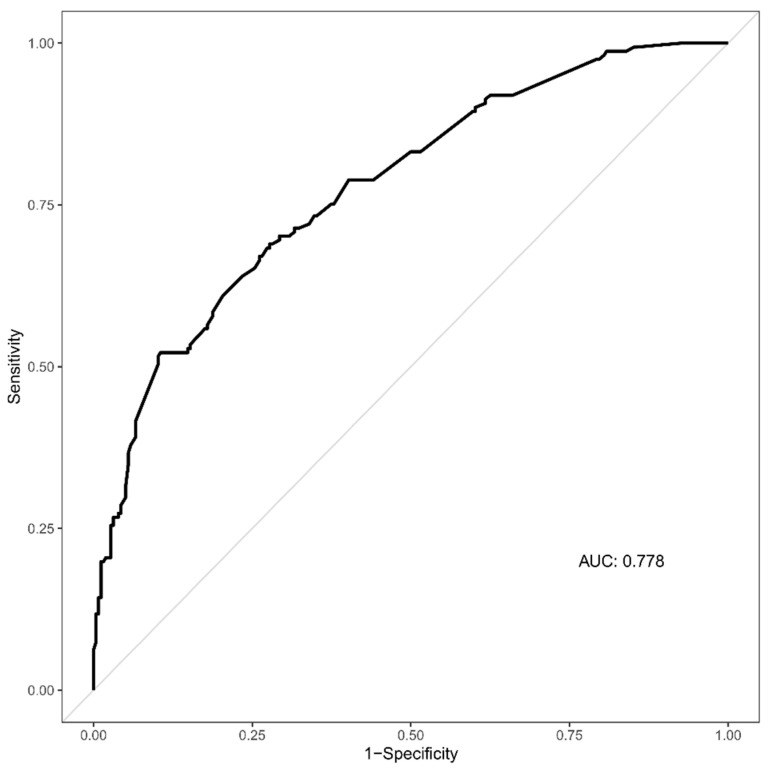
Receiver operating characteristic curve of the multivariable logistic regression predicting epilepsy development in pediatric patients with afebrile seizures.

Table 2 shows the results of the multivariable logistic regression analysis. The age groups of 2–5 years (OR 2.611; 95% CI, 1.263–5.398; *p* = 0.010), 11–15 years (OR 3.138; 95% CI, 1.1469–6.702; *p* = 0.003), and 16–18 years (OR 4.292; 95% CI, 1.677–10.982; *p* = 0.002) were significantly associated with the development of epilepsy. Furthermore, two or more seizures (OR 2.378; 95% CI, 1.324–4.272; *p* = 0.004), a seizure duration of more than 10 min (OR 4.869; 95% CI, 1.579–15.017; *p* = 0.006), lethargy (OR 2.341; 95% CI, 1.134–4.831; *p* = 0.021) and lactate level > 2.27 mg/dL (OR 4.205; 95% CI, 2.585–6.839; *p* < 0.001) were significantly associated with epilepsy development. The VIF ranged from 1.014 to 3.450 for the various risk factors; multicollinearity was not detected. The AUROC of the multivariable logistic regression was 0.778 (95% CI, 0.733–0.823) (Figure 2).

**Figure 3:**
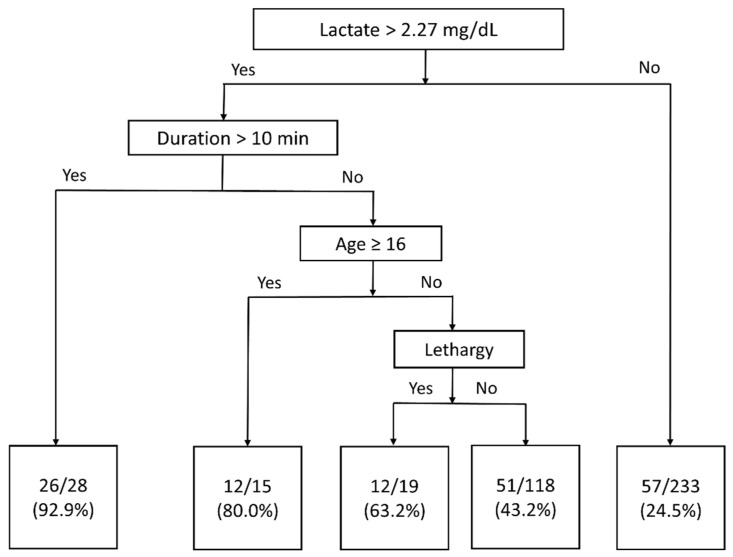
Decision tree for the 417 pediatric patients with new-onset afebrile seizures. The percentages in the bottom boxes represent the risk of epilepsy development.

Figure 3 shows the decision tree drawn to predict epilepsy development in pediatric patients with first afebrile seizure. All 417 pediatric patients with afebrile seizures were included in the decision tree. First, the patients were stratified according to lactate level (threshold = 2.27 mg/dL). Only 24.5% of patients with lactate levels below the threshold developed epilepsy. Second, patients with lactate levels above the threshold were stratified by seizure duration; 92.9% of patients with a seizure duration >10 min developed epilepsy. Third, patients with seizure duration <10 min were stratified by age; 80% of patients aged > 16 years developed epilepsy. Finally, patients aged < 16 years were stratified by lethargy; 63.2% of lethargic patients developed epilepsy, as did 43.2% of those lacking lethargy.

**Figure 4:**
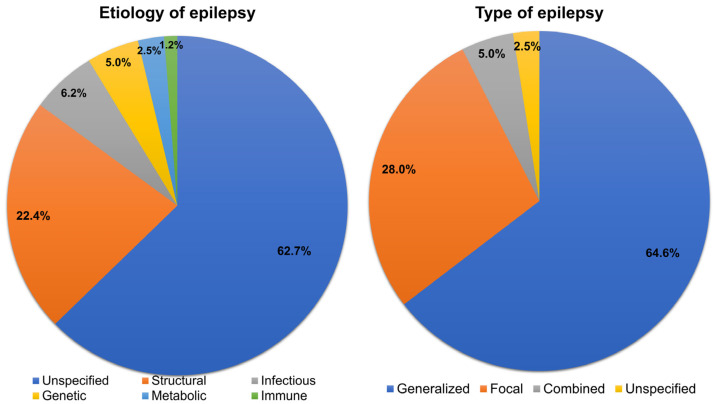
Etiology and type of epilepsy.

Etiologies and types of epilepsy are summarized in Figure 4. The most common etiology of epilepsy was unspecified (*n* = 101, 62.7%), followed by structural (*n* = 36, 22.4%), infectious (*n* = 10, 6.2%), genetic (*n* = 8, 5.0%), metabolic (*n* = 4, 2.5%), and immune (*n* = 2, 1.2%) cause. The most common type of epilepsy was generalized (*n* = 104, 64.6%), followed by focal (*n* = 45, 28.0%), combined (*n* = 8, 5.0%), and unspecified (*n* = 4, 2.5%) type.

## 4. Discussion

In this retrospective multicenter study, older age (2–5, 11–18 years), two or more seizures, longer seizure duration (>10 min), lethargy, and a higher lactate level were significant risk factors for epilepsy development in pediatric patients presenting to EDs with their first afebrile seizure. In previous studies, results were analyzed mainly based on the features of seizures [6,16,17,18], but in this study, various factors such as laboratory results and physical examinations were included. In addition, we investigated the etiologies and types of epilepsy and tried to construct a decision tree for physicians.

According to the ILAE, etiologies of epilepsy are divided into structural, genetic, metabolic, infectious, immune, and unknown etiology and types of epilepsy are categorized as focal, generalized, combined, and unknown type [24]. Our results are in line with a previous study conducted on the etiology of epilepsy; unknown cause was the most common, followed by structural, infectious, and others [25].

Age, as a surrogate of brain maturation, affects the characteristics of seizure disorders in patients with epilepsy [26]. Several authors have investigated the relationship between age at the time of the first afebrile seizure and the risk of epilepsy development, in various populations; the results are still controversial [16,26,27,28,29]. The European Multicenter Epilepsy and Single Seizure (MESS) study, which involved patients of all ages, found that the age of the first unprovoked seizure was not significantly associated with epilepsy development [28]. On the other hand, Ellenberg et al. followed up on 39,270 children for 7 years from birth and reported that children who experienced afebrile seizures when aged 2–5 years were more likely to develop epilepsy [16]. Sartori et al. reported that a first afebrile seizure after the age of 6 years was a significant predictor of epilepsy [29]. Similarly, when comparing patients aged 2–5, 11–15, and 16–18 years to those aged < 2 years, we found that the age of the first unprovoked seizure was significantly associated with epilepsy development.

In our study, longer seizure duration (>10 min) and two or more seizures were significantly associated with epilepsy development. Kim et al. reported that the number of seizures during the presentation and abnormal EEG findings significantly predicted epilepsy development [28]. In their study, patients with two or three seizures constituted the medium-risk group, while those with more than three seizures formed the high-risk group, and those with single seizures constituted the low-risk group [28]. Das et al. found that, among patients with a history of a single seizure, the longer the seizure duration, the greater the risk of epilepsy development [18]. Thus, multiple seizures over a long period may indicate that the patients had a lower threshold for epileptic activity and a higher risk of epilepsy [30].

We found that lethargy in children experiencing their first afebrile seizure was associated with epilepsy development. Few studies have explored the relationship between lethargy and epilepsy [16,31], and the underlying mechanism is yet unclear. Serotonin neurotransmission may give a clue in interpreting our results. The link between serotonin receptors and epilepsy has been suggested by previous studies for many decades [32,33,34]. Moreover, a study found that serotonin neurotransmission (which modulates mood, cognition, sleep, memory, and many physiological processes) was associated with experimentally induced seizures [32]. Lethargy is one of the most common symptoms of serotonin toxicity and seizures are found among patients taking high dose of serotonergic drugs [35]. Children with lethargy may have problems with serotonin neurotransmission which could lead to seizures. In a case report, two children who complained of slow responses and lethargy were finally diagnosed with juvenile myoclonic epilepsy after EEG [31]. However, further studies are required.

Lactate (a non-glucose substrate of cerebral metabolism) is produced in the last step of glycolysis via the action of lactate dehydrogenase [36]. Relationships of temporal lobe epilepsy with the levels of lactate and its transporters have recently been reported [37]. Moreover, Kilic et al. found that the lactate level, as revealed by venous blood gas analysis, predicted seizure recurrence in patients experiencing their first seizures [20]. We also found that a higher lactate level was a significant predictor of epilepsy development in children experiencing their first afebrile seizure.

We also constructed a decision tree to allow physicians to quickly predict the risk of epilepsy development in children experiencing their first afebrile seizure. The predictors can be readily obtained in EDs. Physicians can collaborate with pediatric neurologists to start pediatric epilepsy patients on appropriate treatment such as AEDs.

This study had several limitations. First, as a retrospective study, there was a risk of selection bias. However, this may have been minimal because all three EDs shared one united protocol to manage children with afebrile seizures. Second, the longest follow-up period was only 5 years. However, in most prospective studies assessing whether afebrile seizures predict pediatric epilepsy, the patients typically developed epilepsy within 1 year [6,16]. Thus, we identified most of the patients with epilepsy. Third, the results may not generalize to other races or countries. Fourth, since this study was conducted on patients visiting the ED, seizures in which symptoms may not be noticeable, such as absence seizure, may have been missing. A large-scale prospective study is required to identify predictors of epilepsy in pediatric afebrile seizure patients.

## 5. Conclusions

A lactate level above 2.27 mg/dL, lethargy, seizure duration of more than 10 min, two or more seizures, and older age of first unprovoked seizure (2–5, 11–18 years) were significant predictors of epilepsy development in children with new-onset afebrile seizures. Physicians must be aware that additional seizures are possible in children with such findings and should provide appropriate treatment and follow-up to them.

## Figures and Tables

**Table 1:** Comparison of baseline characteristics between the two groups.

Variables	Non-Epilepsy (*n* = 256)	Epilepsy (*n* = 161)	*p*-Value
Age of first unprovoked seizure, years	6 [1.7–10]	7 [3–13]	0.015
Sex, *n* (%)			0.448 *
Female	113 (44.14)	78 (48.45)	
Male	143 (55.86)	83 (51.55)	
Medical history			
IUP, weeks	38 [37–39.1]	38 [37–39.6]	0.089
Birth weight, kg	3 [2.6–3.3]	3 [2.7–3.3]	0.456
C-sec history, *n* (%)	113 (44.14)	70 (43.48)	0.975 *
Familial history, *n* (%)	35 (13.67)	27 (16.77)	0.469 *
NICU history, *n* (%)	17 (6.64)	16 (9.94)	0.304 *
Seizure features			
No. of seizures	1 [1–1]	1 [1–2]	<0.001
Duration, min	3 [1–5]	5 [2–10]	<0.001
Todd’s paralysis, *n* (%)	4 (1.56)	10 (6.21)	0.022 *
Postictal confusion (%)	160 (62.50)	120 (75.53)	0.147 *
Seizure type, *n* (%)			0.340 *
GTC	230 (89.84)	140 (86.96)	
Focal	25 (9.77)	18 (11.18)	
Secondary GTC	1 (0.39)	3 (1.86)	
Symptoms, *n* (%)			
Headache	20 (7.81)	15 (9.32)	0.720 *
Vomiting	57 (22.27)	32 (19.88)	0.648 *
Diarrhea	26 (10.16)	6 (3.73)	0.027 *
POI	24 (9.38)	9 (5.59)	0.227 *
Lethargy	26 (10.16)	30 (18.63)	0.020 *
Physical examinations, *n* (%)			
Dehydrated tongue	13 (5.08)	1 (0.62)	0.029 *
Neck stiffness	1 (0.39)	3 (1.86)	0.218 **
Babinski sign	0 (0)	2 (1.24)	0.149 **
Laboratory results			
pH	7.36 [7.3–7.4]	7.33 [7.2–7.4]	<0.001
Bicarbonate, mmol/L	23.6 [20.9–25.7]	23.5 [21.2–26.1]	0.415
Lactate, mg/dL	1.9 [1.4–2.6]	2.7 [1.8–4.9]	<0.001
WBC, 10^3^/µL	8.59 [6.8–10.9]	8.79 [6.9–12.8]	0.113
Hb, g/dL	12.8 [12.0–13.4]	12.8 [12.1–13.6]	0.549
PLT, 10^3^/µL	297.5 [256–360]	293 [239–344]	0.268
BUN, mg/dL	11.6 [9.5–13.8]	11 [9.0–13.2]	0.041
Cr, mg/dL	0.5 [0.4–0.6]	0.5 [0.4–0.7]	0.004
Glucose, mg/dL	100 [91–110]	108 [97–127]	<0.001
Albumin, g/dL	4.6 [4.4–4.8]	4.5 [4.4–4.7]	0.037
Bilirubin, mg/dL	0.31 [0.2–0.5]	0.36 [0.2–0.5]	0.281
AST, U/L	29 [23–38]	28 [22–35]	0.439
ALT, U/L	14 [11–19.3]	14 [11–20]	0.685
Sodium, mmol/L	139 [138–140]	140 [138–141]	0.005
Chloride, mmol/L	103 [101.8–104]	103 [102–105]	0.058
Total calcium, mg/dL	9.6 [9.2–10]	9.2 [8.9–9.6]	<0.001
CRP, mg/L	1.6 [0.4–4.0]	0.8 [0.3–3.8]	0.033

Categorical variables are presented as numbers (percentage) and were compared using the * Pearson chi-squared test or ** Fisher’s exact test. Continuous variables are presented as median [interquartile range] and were compared with the Mann-Whitney U test. Abbreviations: IUP, intrauterine pregnancy; C-sec, cesarean section; NICU, neonatal intensive care unit; GTC, generalized tonic-clonic; POI, poor oral intake; pH, potential of hydrogen; WBC, white blood cell; Hb, hemoglobin; PLT, platelet; BUN, blood urea nitrogen; Cr, creatinine; AST, aspartate aminotransferase; ALT, alanine transaminase; CRP, C-reactive protein.

**Table 2:** Multivariable logistic regression analyses of risk factors for the possibility of transition to epilepsy in pediatric afebrile seizure patients.

Variables	Multivariable		
	OR (95% CI)	*p*-Value	VIF
Age of first unprovoked seizure, years			
<2	1		
2–5	2.611 (1.263–5.398)	0.010	1.977
6–10	1.897 (0.917–3.924)	0.084	1.936
11–15	3.138 (1.469–6.702)	0.003	1.806
16–18	4.292 (1.677–10.982)	0.002	1.553
Seizure features			
Counts ≥ 2	2.378 (1.324–4.272)	0.004	1.097
Duration, min			
<1	1		
1–5	1.629 (0.660–4.024)	0.289	3.450
6–10	1.342 (0.458–3.939)	0.592	2.564
>10	4.869 (1.579–15.017)	0.006	2.489
Todd’s paralysis	2.289 (0.569–9.205)	0.243	1.081
Symptoms			
Diarrhea	0.426 (0.134–1.352)	0.148	1.181
Lethargy	2.341 (1.134–4.831)	0.021	1.122
Physical examinations			
Dehydrated tongue	0.123 (0.010–1.566)	0.107	1.155
Laboratory results			
Cr > 1.21, mg/dL	0.444 (0.030–6.478)	0.552	1.064
Glucose, mg/dL			
<50	2.445 (0.174–34.407)	0.508	1.014
50–144	1		
>144	1.825 (0.706–4.714)	0.552	1.064
Lactate > 2.27, mg/dL	4.205 (2.585–6.839)	<0.001	1.160

Abbreviations: OR, odds ratio; CI, confidence interval; VIF, variance inflation factor; Cr, creatinine.

## Data Availability

Not applicable.
